# Meeting the UN Sustainable Development Goals with Mechanochemistry

**DOI:** 10.1002/anie.202414745

**Published:** 2024-11-06

**Authors:** Jasna Alić, Moritz‐Caspar Schlegel, Franziska Emmerling, Tomislav Stolar

**Affiliations:** ^1^ BAM Federal Institute for Materials Research and Testing Richard-Willstätter-Strasse11 12489 Berlin Germany

**Keywords:** mechanochemistry, sustainable development goals, environmental remediation, climate action, energy efficiency

## Abstract

Chemistry traditionally relies on reactions in solution, but this method is increasingly problematic due to the scale of chemical processes and their economic and environmental impact. Handling residual chemical waste, including solvents, incurs significant costs and environmental pressure. Conversely, novel chemical approaches are needed to address pressing societal issues such as climate change, energy scarcity, food insecurity, and waste pollution. Mechanochemistry, a sustainable chemistry discipline that uses mechanical action to induce chemical reactivity without bulk solvents, is a hot topic in academic research on sustainable and green chemistry. Given its fundamentally different working principles from solution chemistry, mechanochemistry offers more efficient chemical processes and the opportunity to design new chemical reactions. Mechanochemistry has a profound impact on many urgent issues facing our society and it is now necessary to use mechanochemistry to address them. This Minireview aims to provide a guide for using mechanochemistry to meet the United Nations (UN) Sustainable Development Goals (SDGs), thereby contributing to a prosperous society. Detailed analysis shows that mechanochemistry connects with most UN SDGs and offers more cost‐efficiency than other approaches together with a superior environmental performance.

## Graphical abstract

This minireview explores how mechanochemistry can address the United Nations Sustainable Development Goals. The potential of mechanochemistry to address climate change, energy scarcity, food insecurity and waste pollution by providing more efficient and environmentally friendly chemical processes is highlighted. Key advances and applications in sustainable chemistry are discussed.

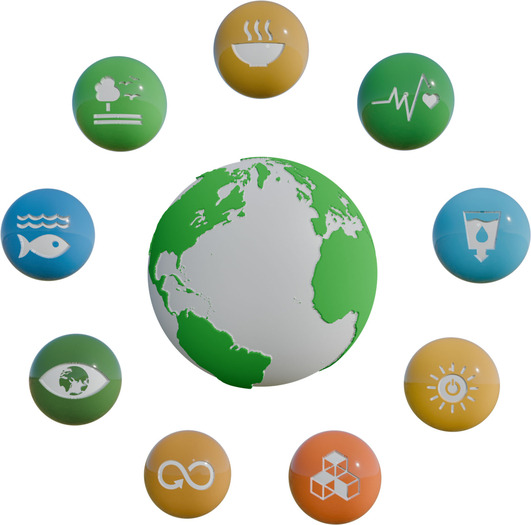

## From an Ancient Tool to a Modern Discipline

The term “mechanochemistry” was coined by Wilhelm Ostwald in 1919.^[1]^ He defined mechanochemistry as a branch of chemistry that studies chemical and physical changes of substances in any aggregation state resulting from mechanical action. Different definitions of mechanochemistry are currently used.^[2]^ However, mechanochemistry has been around since ancient times and people have used it to start a fire by rubbing two stones together or creating friction with a wooden stick.^[3]^ Grinding has also been used for millennia to prepare food and perform (al)chemical reactions.^[4]^ The first documented mechanochemical reaction was reported in the fourth century BC in the book of Theophrastus, a student of Aristotle. He described the reduction of cinnabar to elemental mercury and chalcocite in a mortar and pestle made of copper using catalytic amounts of vinegar.^[5]^ Notably, the need for copper and vinegar is not immediately apparent, the reaction does not work in stone or iron equipment, and it is inefficient when water is used as a liquid additive. However, performing chemistry by mechanical action has historically been overshadowed by performing it in solution, perhaps influenced by “*no coopora nisi fluida*”, meaning “no reaction occurs in the absence of solvent”.^[6]^

**Figure 1:**
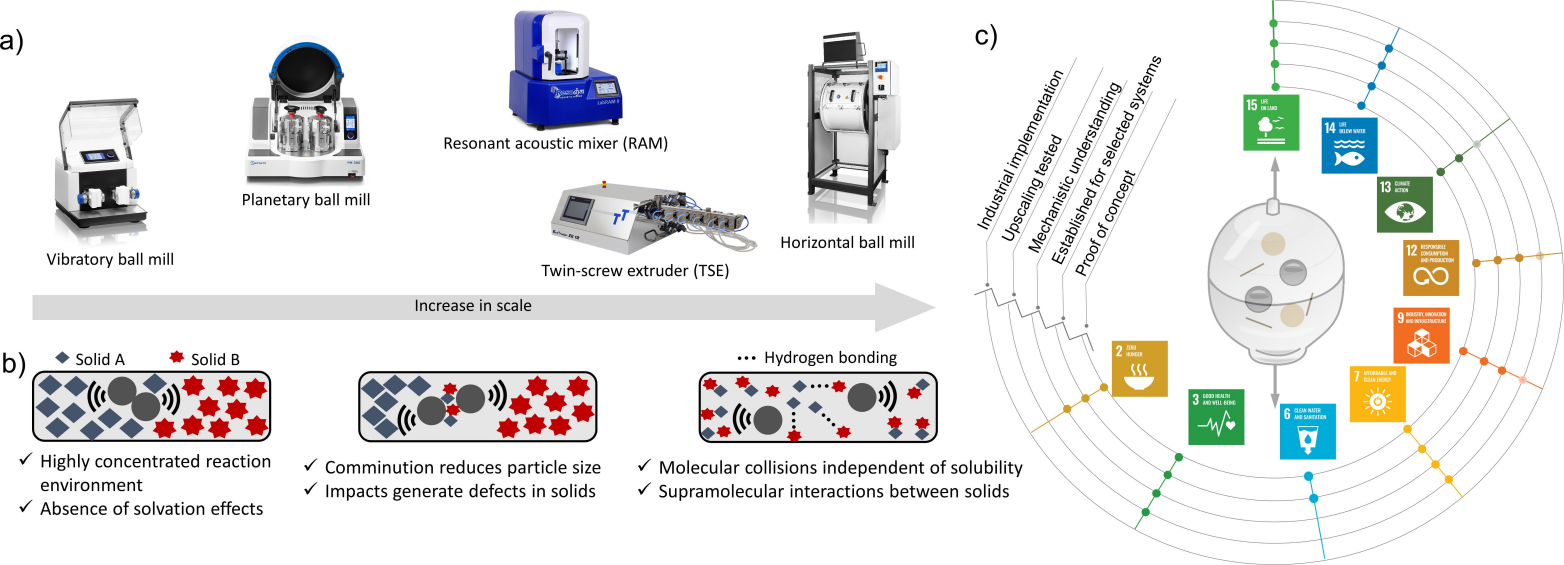
a) Typical devices employed in mechanochemistry research. b) Specific working principles that differentiate mechanochemistry from solution chemistry. c) Potential and proven possibilities to support the United Nations Sustainable Development Goals (SDGs). The concentric lines represent different levels of development.

The recent renaissance of mechanochemistry is a result of the green chemistry movement,^[7]^ as mechanochemistry fits well with the 12 principles of green chemistry.^[8]^ Manual grinding experiments are now performed in mills, extruders, and resonant acoustic mixers that generate different types of mechanical forces (Figure 1a).^[9]^ Mechanochemistry as a synthetic tool has found application in all types of chemistry,[^10^, ^11^, ^12^, ^13^, ^14^, ^15^, ^16^, ^17^, ^18^, ^19^] and various types of in situ monitoring methods provided insights into mechanochemical processes kinetics, and mechanisms.[^20^, ^21^] The latest developments in chemical reactivity are new approaches combining mechanochemical action with other energy sources. These include thermo‐mechanochemical, photo‐mechanochemical, sono‐mechanochemical, and electro‐mechanochemical methods, which offer new possibilities for chemical reactivity.^[22]^

A closer look at the working principles of mechanochemistry reveals the differences to the traditional solution chemistry (Figure 1b) and helps to understand why mechanochemistry usually performs better than the latter. Mechanochemistry is exceptionally effective at facilitating two fundamental requirements for chemical reactions: promoting molecular collisions and providing sufficient activation energy. The recent discovery of non‐covalent interactions between reagents that control the formation of covalent bonds indicates that these interactions play an important role in many mechanochemical reactions.[^23^, ^24^, ^25^] This is in sharp contrast to solution reactions, where such non‐covalent interactions are often hindered or undetectable.

Moreover, comminution, impacts, and mixing effects facilitate overcoming activation barriers that are typically associated with solid‐state reactions. Overall, mechanochemistry not only represents a greener approach to chemical processes but also offers the opportunity to redesign these processes at a fundamental level. In the following paragraphs, we will analyse how mechanochemistry relates to individual SDGs (Figure 1c) that are directly related to chemistry or where chemistry plays a significant role.^[26]^ In general, the UN SDGs were adopted in 2015 by all UN member states after decades of work by several countries and the UN.^[26]^ The SDGs offer a blueprint for peace and prosperity for the people and the planet. Through global partnerships, the SDGs aim to reduce inequalities, neutralise negative anthropogenic effects, and ensure sustainable development that does not come at the cost of environmental and ecological degradation.

## SDG 2: Zero Hunger

There is a significant opportunity for mechanochemistry to make a substantial contribution to SDG Number 2: Zero Hunger. This goal is focused on ending hunger, achieving food security, improving nutrition, and promoting sustainable agriculture. The applications of mechanochemistry in achieving SDG 2 include the development of more efficient fertilisers, the enhancement of nutrient extraction from food sources, improving food preservation and packing techniques, and the creation of novel food processing methods.

Ammonia is the principal precursor of nitrogen‐based fertilisers and is among the most produced industrial chemicals globally. The Haber–Bosch process, developed in the early 20^th^ century, is currently the most widely used method for synthesising ammonia.^[27]^ The Haber–Bosch process combines nitrogen and hydrogen in a catalytic process which requires extremely high temperatures (400–500 °C) and pressures (100–200 atm). It accounts for 1 % of the world‘s total energy production and about 1.4 % of global CO_2_ emissions.^[28]^ Mechanochemical approaches have shown promise as an alternative to the Haber–Bosch process by enabling ammonia production at room temperature.[^29^, ^30^, ^31^] Interestingly, even a blade shaft could achieve ammonia synthesis through mechanical stress.^[29]^ In a recent study, successful production of ammonia through ball milling has been reported.^[32]^ That approach enabled ammonia synthesis under mild conditions of 45 °C and 1 bar in the presence of iron powders, providing a novel and sustainable alternative. Researchers were able to produce ammonia at higher yields calculated on the volume of the gas released (82.5 % vol.), with a lower total energy requirement of 4.5*10^12^ J compared to the Haber–Bosch process (39*10^12^ J).

**Figure 2:**
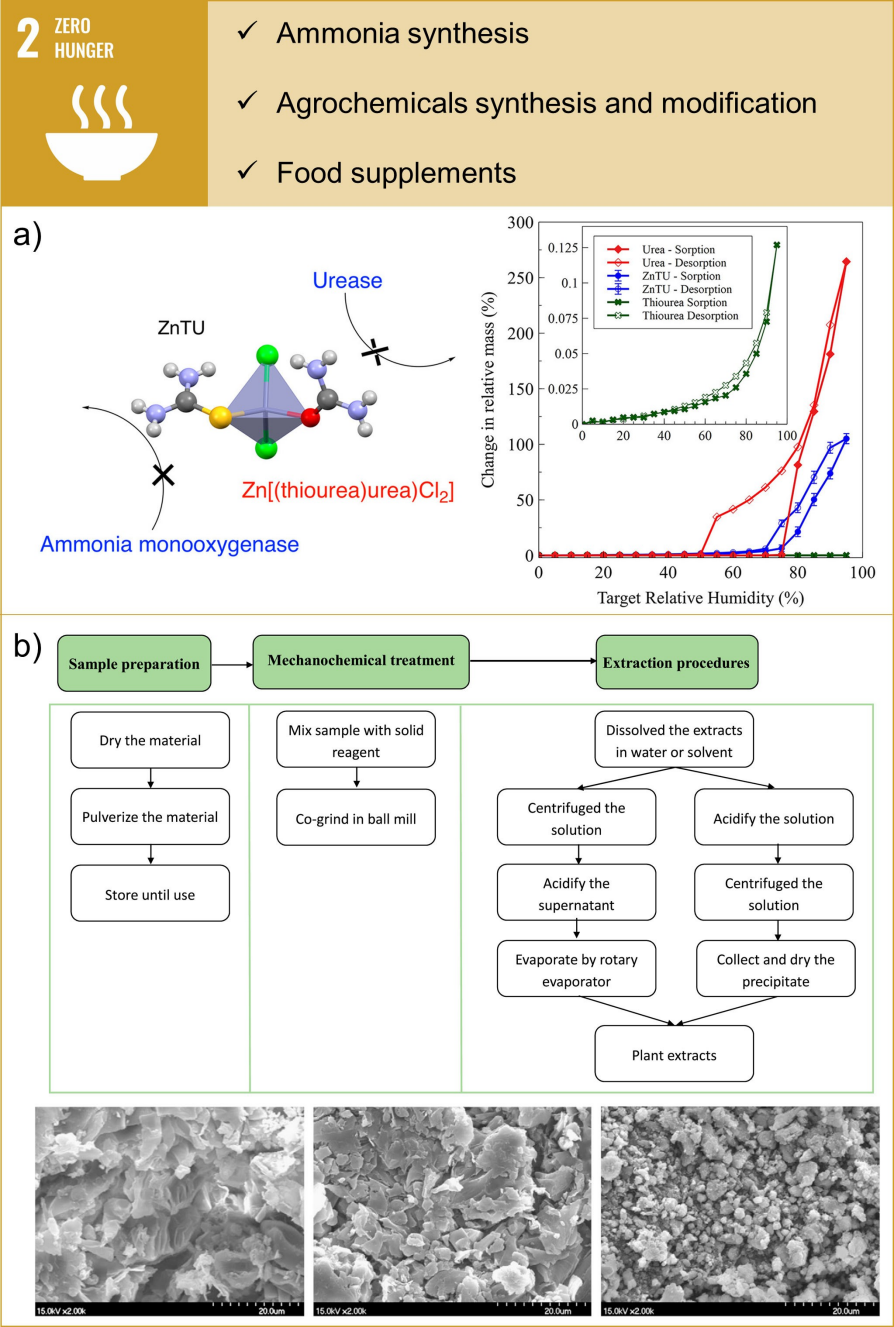
Examples of contributions of mechanochemistry towards SDG 2: Zero Hunger. a) Enhanced efficiency fertilisers: Ternary Zn(II)‐thiourea‐urea ionic cocrystal [Zn(thiourea)(urea)Cl_2_], (ZnTU) acting as urease and ammonia monooxygenase inhibitor; adsorption/desorption branches of RH on urea, ZnTU, and thiourea. Reprinted with permission from Ref. [34]. Copyright 2019 American Chemical Society. b) Plant extracts: Details of the mechanochemical assisted extraction (MAE) process and SEM pictures of Hibiscus mutabilis samples after different pretreatments: shattered (left), superfine powder (middle), and mechanochemically treated sample (right). Reprinted with permission from Ref. [35]. Copyright 2017 Elsevier.

The careful use of agrochemicals, including fertilisers, herbicides, and fungicides, is vital for the long‐term viability of food production. However, these chemicals are susceptible to hydrolysis and loss of nitrogen to the atmosphere. This can be mitigated through the controlled release and enhanced stability of these chemicals under environmental conditions. Mechanochemistry can facilitate the development of enhanced efficiency fertilisers (EEFs), such as urea cocrystals, which exhibit higher nitrogen use efficiency and lower environmental impact when compared to conventional fertilisers.^[33]^ In a recent publication, a ternary Zn(II)‐thiourea‐urea ionic cocrystal (ZnTU, [Zn(thiourea)(urea)Cl_2_]) has been reported, offering a new approach to soil nitrogen management by inhibiting enzymes responsible for reducing urea‐based nitrogen fertilisation efficiency (see Figure 2a).^[34]^ The cocrystals can enhance nitrogen management by inhibiting the conversion of ammonia to nitrite via ammonia monooxygenase. This limits the formation of nitrate, which is prone to leaching into groundwater, and promotes sustainable soil fertilisation practices. Together with the reduced solubility of ZnTU compared to pure urea its potential for controlled release of nutrients in soil allows for more efficient nutrient uptake by plants over time.

In the context of nutrient extraction and food processing, mechanochemical assisted extraction (MAE) has emerged as a novel, efficient, and eco‐friendly technology for extracting valuable compounds from food sources. This method offers several advantages over conventional extraction techniques, including reduced solvent use, lower energy consumption, and higher extraction efficiency.^[35]^ MAE is particularly beneficial in extracting bioactive compounds, such as antioxidants and essential oils, from plant materials. By improving the extraction of these nutritionally valuable compounds, mechanochemistry can contribute to enhancing the nutritional quality of food products. For example, MAE can be used to extract flavonoids from bamboo leaves.^[36]^ Planetary ball milling of shredded bamboo leaves with Na_2_CO_3_ (12 wt %) and Na_2_B_4_O_7_ ⋅ 10H_2_O (2 wt %) for 10 min resulted in particle size reduction and improved hydrophilicity of flavonoids. Enhanced extraction using MAE was found to be superior to heat reflux extraction and alkaline extraction, which yielded lower recoveries of total flavonoids compared to MAE. Similarly, planetary ball milling of *Ginkgo biloba* leaves with NaHCO_3_ resulted in conversion of flavonoids and terpene trilactone to their water‐soluble salts.^[37]^ Thus, MAE enabled the use of water as the only extracting solvent and showed higher recovery rates compared to heat reflux extraction. Another example of using MAE for the transformation of bioactive compounds to water‐soluble salts is the extraction of magnolol from *Magnolia officinalis*.^[38]^

Mechanochemistry also holds promise for developing sustainable packaging solutions for the food industry. Packaging plays a crucial role in the preservation and distribution of food, but traditional packaging materials often contribute to environmental pollution. Mechanochemical processes can create biodegradable and compostable packaging materials from agricultural waste products.^[39]^ For example, nanocellulose was isolated from the abundant shrub plant *Amorpha fruticose* by combined grinding and homogenisation.^[40]^ The isolated nanocellulose exhibited fine structures with a diameter of 10 nm and retained crystal structure and hydroxy groups of cellulose. Mechanochemically obtained nanocellulose showed higher thermal stability compared to natural wood. Moreover, high efficient process of oxidation of cellulose to useful dialdehyde cellulose has been developed using vibratory ball milling.^[41]^ Ball milling cellulose, water, and sodium metaperiodate as oxidant, resulted in maximum aldehyde content of 8 mmol g^−1^ with a periodate/cellulose molar ratio of 1.25, a milling time of 2 min, and a resting time of 8 h. Furthermore, mechanochemical processes can transform agricultural waste and biomass into value‐added chemicals, materials, and fuels.[^42^, ^43^] This approach has the potential to reduce waste, create new income streams for farmers, and provide sustainable alternatives to fossil‐based products.

## SDG 3: Good Health & Well‐Being

**Figure 3:**
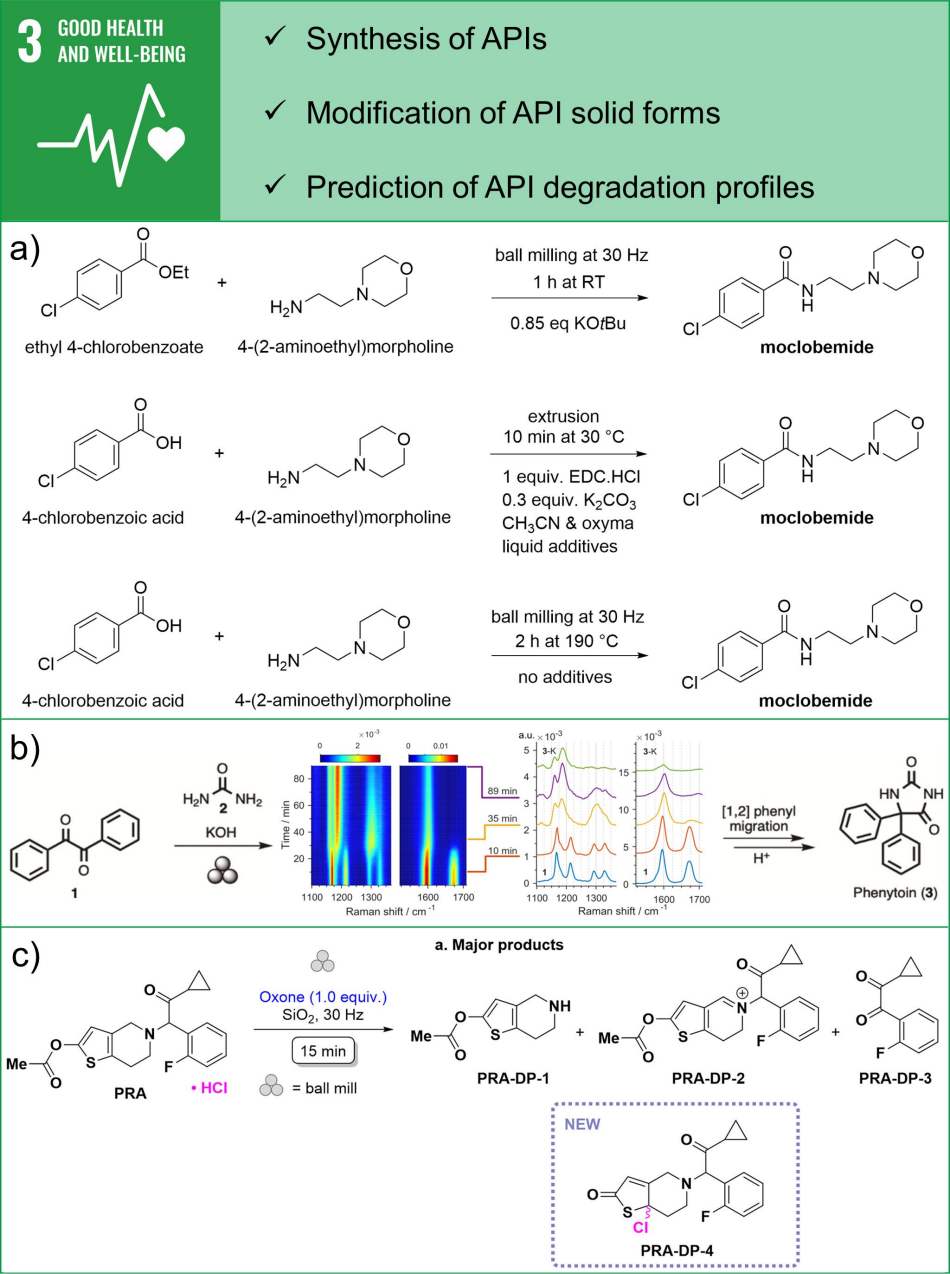
Examples of contributions of mechanochemistry towards SDG 3: Good Health & Well‐Being. a) Different mechanochemical approaches for amide bond formation and synthesis of moclobemide API. b) In situ monitoring by Raman spectroscopy enables elucidation of the reaction mechanism by observation and isolation of intermediates in mechanochemical synthesis of phenytoin, a WHO essential medicine. Adapted under the terms of CC BY license from Ref. [48]. c) Mechanochemical oxidative degradation of prasugrel hydrochloride API for obtaining a fast degradation profile. Adapted with permission from Ref. [54]. Copyright 2023 American Chemical Society.

Important for promoting well‐being and enabling healthy lives is the mechanochemical synthesis of active pharmaceutical ingredients (APIs), their solid form modifications to improve drug efficiency, and the prediction of API degradation profiles.[^44^, ^45^] The formation of the amide bond is the most used chemical transformation in drug discovery and traditional preparation of amides uses excess solvents and stoichiometric ratios of coupling reagents, thus yielding large amounts of waste, and resulting in a poor atom economy. However, ball milling enables solvent‐free amidation without activating agents when amines are coupled with esters.^[46]^ The approach uses substoichiometric amounts of KOtBu solid base and applies to a broad substrate scope which includes aryl and alkyl ester substrates together with secondary and primary amines. Several active pharmaceutical ingredients (APIs) such as moclobemide were prepared using this methodology and scaled up to a gram scale (Figure 3a top). Furthermore, reactive extrusion is a powerful tool to synthesise amide‐based APIs and is especially energy‐efficient if the reaction times are short.^[47]^ This was demonstrated in the near‐quantitative preparation of moclobemide, which needed 5 min of extrusion at 30 °C using the combination of solid and liquid reagents (Figure 3a centre). Although coupling reagents were needed to activate the carboxylic acid, the use of solvents was drastically reduced in a continuous liquid‐assisted extrusion process. Alternatively, direct amidation of carboxylic acids and amines without solvents and activating reagents has been achieved by a thermo‐mechanochemical approach.^[25]^ Quantitative synthesis of moclobemide with an almost ideal atom economy was enabled by milling under high temperatures and eliminating water from the reaction mixture (Figure 3a bottom). Additionally, in situ reaction monitoring by synchrotron PXRD and crystallographic characterisation revealed that the formation of moclobemide is preceded by the formation of ammonium carboxylate salt.

Another example that shows valuable insights from in situ monitoring methodologies is a detailed mechanistic study of the mechanochemical preparation of phenytoin, a World Health Organisation (WHO) essential medicine.^[48]^ In situ monitoring by Raman spectroscopy helped to elucidate the complex reaction mechanism, which had been elusive in solution synthesis, by detecting intermediates that were subsequently isolated (Figure 3b). It has been shown that a one‐pot mechanochemical transformation of benzil, urea, and KOH into phenytoin involves diol, imidazolone, and ketal intermediates.

Paracetamol, another WHO essential medicine, has recently been synthesised by bead milling.^[49]^ The described bead milling batch process used a large number of small beads (Y‐stabilised ZrO_2_, 5 mm in diameter). Paracetamol was obtained in 88 % conversion from *p*‐hydroxyacetophenone oxime, oxalic acid, and *p*‐tosylimidazole after 30 min of bead milling at 6000 rpm using 80 v/v % bead filling rate and 0.6 ratio of reactants and beads. Importantly, microwave plasma‐atomic emission spectroscopy demonstrated below limits product contamination coming from ZrO_2_ and Y_2_O_3_, an important aspect for the industrial application of bead milling technology that can also be operated in a continuous mode.

In this sense, continuous mechanochemical processing offers advantages such as increased product output compared to batch processes. It has been shown that twin screw extrusion (TSE) can be used for the selective synthesis of API cocrystal polymorphs simply by changing the liquid additives.^[50]^ Specifically, cocrystal polymorph I composed of vitamin B3 (nicotinamide) and vitamin C (ascorbic acid) is obtained by TSE when ethanol is used as a liquid additive, whereas cocrystal polymorph II is prepared when using methanol. Both cocrystal polymorphs were shown to retain the antioxidant activity of vitamin C and demonstrated excellent direct tableting properties. In addition, the same type of vitamin B3 and C cocrystal polymorph selectivity based on methanol and ethanol liquid additives has been reported by resonant acoustic mixing (RAM).^[51]^ Similarly, an approach based on dual asymmetric centrifugal mixing without grinding media has been used for selective scale‐up and discovery of model pharmaceutical cocrystal forms using liquid additives.^[52]^ A notable example of using TSE is the continuous solvent‐free synthesis of nitrofurantoin and dantrolene APIs with high space‐time yields and no need for post‐synthesis workup.^[53]^

Another recent example of the value of mechanochemistry is the prediction of drug stability, a key step before a drug can be marketed, which typically involves time‐consuming and resource‐intensive stability testing.^[54]^ Instead, mechanochemical oxidative degradation is an excellent alternative that reliably predicts drug stability and degradation profiles. For example, rapid ball milling of prasugrel hydrochloride with Oxone and SiO_2_ for only 15 minutes at room temperature yielded four degradation products, one of which was discovered for the first time, demonstrating the full power of mechanochemistry (Figure 3c).

## SDG 6: Clean Water and Sanitation

**Figure 4:**
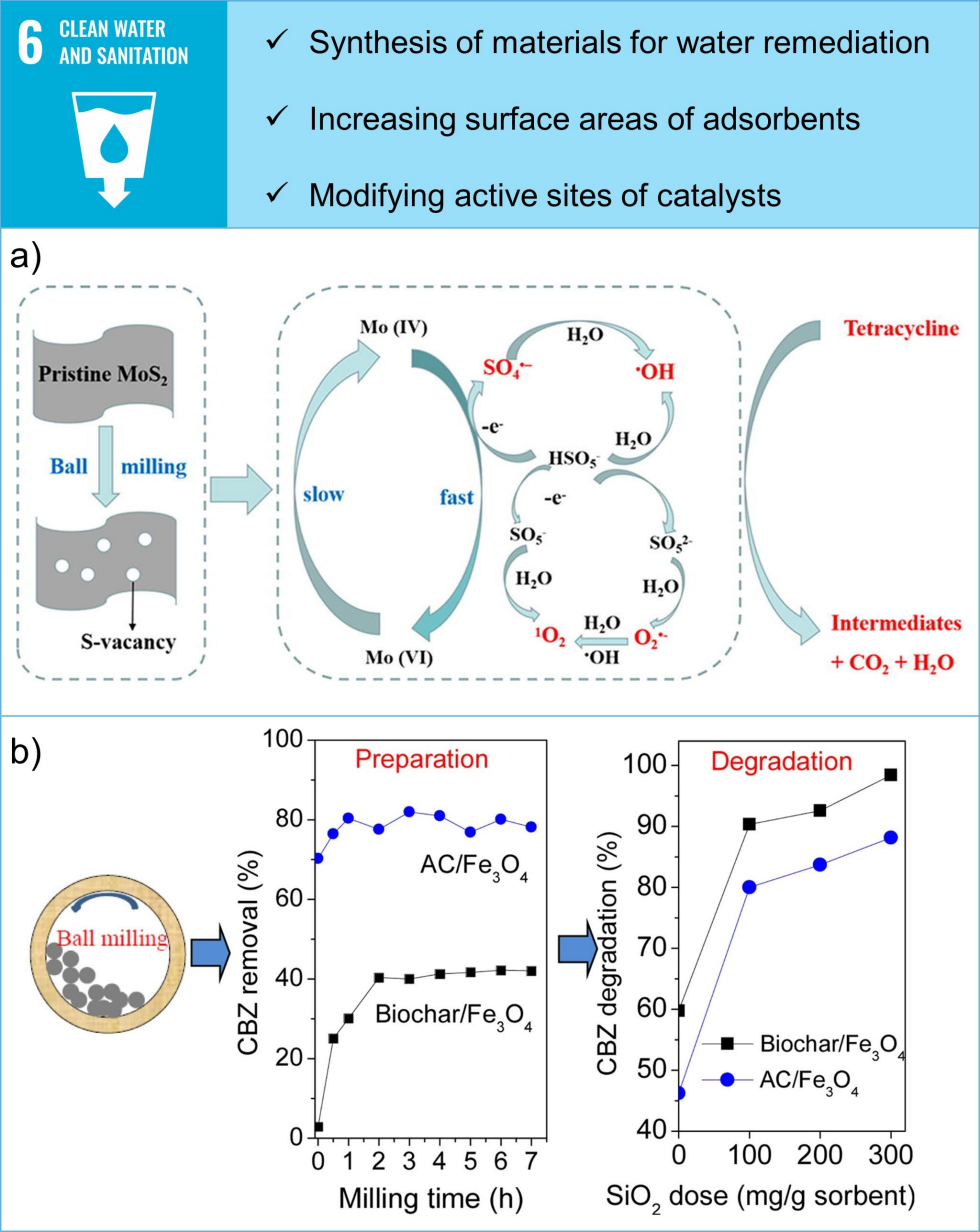
Examples of contributions of mechanochemistry towards SDG 6: Clean Water and Sanitation. a) Ball milling improves catalytic performance of MoS_2_ catalyst for degradation of tetracycline antibiotic. Reprinted with permission from Ref. [56]. Copyright Elsevier 2022. b) Ball milling enables the preparation of ultrafine biochar/Fe_3_O_4_ and activated carbon/Fe_3_O_4_ composites for adsorption and subsequent degradation of carbamazepine and tetracycline from water. Reprinted with permission from Ref. [60]. Copyright Elsevier 2016.

Mechanochemistry contributes to SDG 6 by green synthesis of advanced materials for water decontamination.^[55]^ It also improves their performance by increasing surface areas and modifying active sites through comminution, introduction of defects, or amorphisation. Mechanochemically prepared functional materials find applications in the remediation of antibiotics,^[56]^ heavy metals,[^57^, ^58^, ^59^] pharmaceuticals,^[60]^ dyes,^[61]^ and chlorinated compounds.^[62]^ For instance, ball milling improves the catalytic performance of MoS_2_, a known transition metal dichalcogenide catalyst, in the activation of peroxymonosulfate (PMS) for the degradation of tetracycline (TC) antibiotic.^[56]^ Mechanochemical pretreatment of MoS_2_ increases the number of S‐vacancies and exposes more Mo (IV) active sites on the surface, thus accelerating the reaction with PMS and subsequent TC degradation (Figure 4a).

Furthermore, mechanochemistry remediates heavy metals contaminated wastewater by enabling reactions between insoluble reagents and metals. Using this approach, almost quantitative removal of Pb(II) ions from lead‐zinc contaminated water is achieved by wet grinding contaminated water samples with CaCO_3_.^[57]^ Mechanochemical treatment alters the highly stable CaCO_3_ structure and creates active surfaces that facilitate the reaction with Pb(II) ions in water. More importantly, the mechanochemical activation of insoluble CaCO_3_ avoids using conventional chemical precipitation methods with hydroxides or sulphides, which produce strong alkaline solutions or complex polysulfide complexes.[^63^, ^64^]

Another challenge with traditional water decontamination technologies is the difficulty in separating used adsorbents from contaminated sources or the low adsorption capacity of otherwise promising adsorbents. Mechanochemistry provides an alternative as demonstrated for biochar, a cheap and effective adsorbent with limited commercial use due to lower adsorption capacity compared to active carbon.^[60]^ Ball milling enables simple and fast preparation of ultrafine biochar/Fe_3_O_4_ and activated carbon/Fe_3_O_4_ composites for adsorption of carbamazepine and tetracycline from water. In addition, pharmaceuticals adsorbed on hybrid biochar composites are almost quantitatively degraded in the case of carbamazepine after 3 hours of ball milling with the addition of SiO_2_. Finally, the magnetic properties of Fe_3_O_4_ allowed easy separation of spent adsorbents with adsorbed drugs from water, confirming the multiple benefits of the mechanochemical approach.

## SDG 7: Affordable and Clean Energy

Mechanochemistry has emerged as a promising approach to support the goals of Sustainable Development Goal 7 (SDG 7)—Affordable and Clean Energy. By enabling more sustainable and energy‐efficient processes for producing chemicals, materials, and fuels, mechanochemistry can help advance the transition to clean energy technologies. Mechanochemistry has been widely applied for the synthesis of various materials for energy storage and conversion applications, such as batteries and photovoltaics, in a sustainable and scalable manner.^[65]^

In the field of battery materials, mechanochemical synthesis has been used to produce cathode materials, anode materials, and solid electrolytes for lithium‐ion and sodium‐ion batteries.[^66^, ^67^] Interestingly, mechanochemical synthesis of the Li‐rich anti‐perovskite (Li_2_Fe)SO can be performed as a one‐step process that could be scaled up and optimised increasing the ball‐to‐powder ratio.^[67]^ Lithium transition metal oxides with spinel and layered structures (e.g., LiMn_2_O_4_, LiCoO_2_, LiNiO_2_) are synthesised mechanochemically as high‐performance cathode materials.^[68]^ Conversion‐type anode materials like metal oxides and sulphides (e.g., Fe_3_O_4_, SnO_2_, FeS_2_) are produced via mechanochemical routes, often with reduced particle size and improved electrochemical properties.[^65^, ^69^] Solid electrolytes like Li_3_YCl_6_, Li_6_PS_5_X (X=Cl, Br, I), and Na_11_Sn_2_PSe_12_ are synthesised by ball milling, enabling control over cation disorder and achieving high ionic conductivities.^[70]^

**Figure 5:**
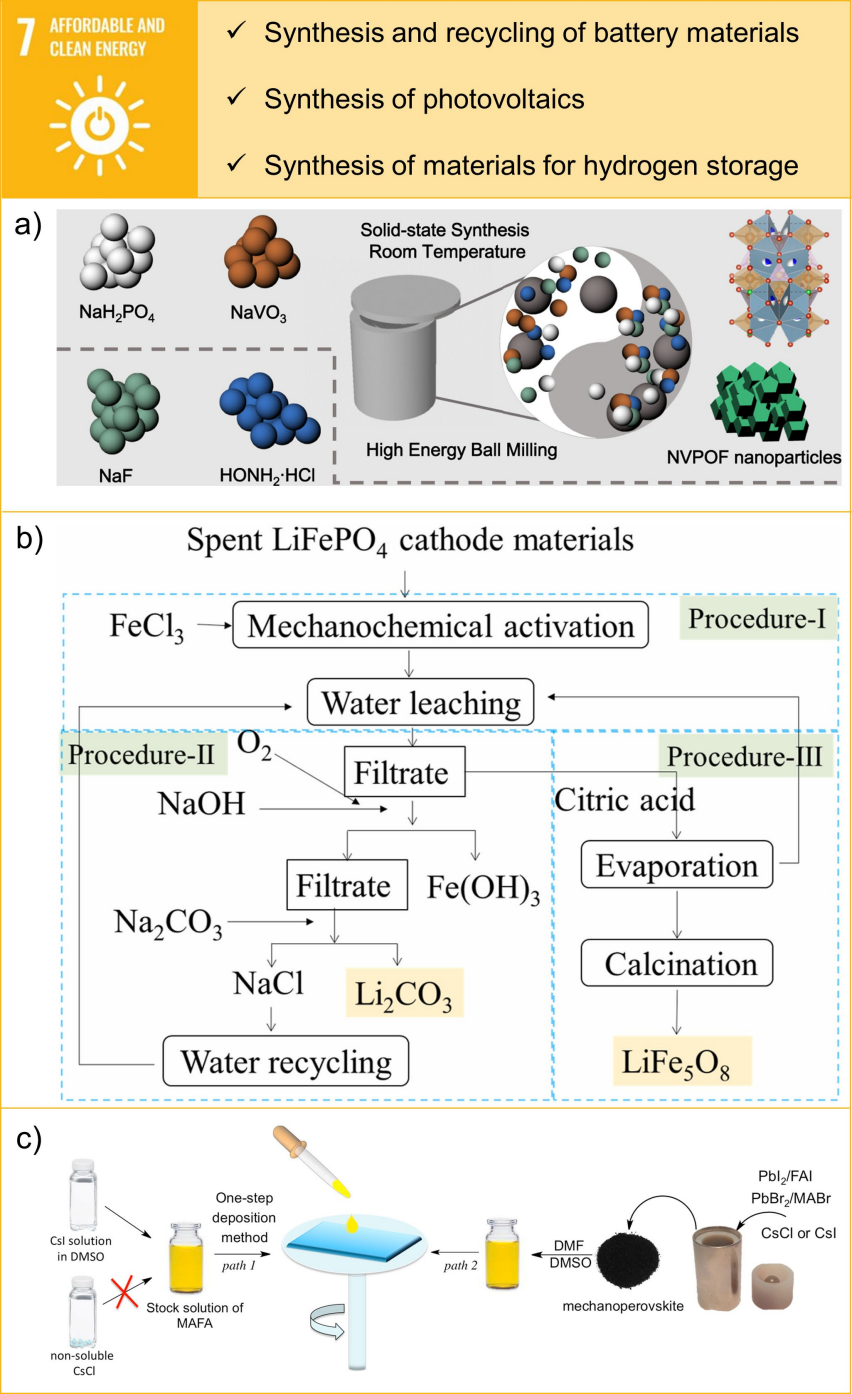
Examples of contributions of mechanochemistry towards SDG 7: Affordable and Clean Energy. a) The mechanochemical synthesis of Na_3_(VOPO_4_)_2_F nanoparticles starting from NaVO_3_. Reprinted with permission from Ref. [66]. Copyright 2021 Springer Nature. b) Flow chart detailing the tandem approach for the selective lithium recovery and lithium ferrite preparation. Reprinted with permission from Ref. [71]. Copyright, Elsevier, 2023. c) Scheme of the one‐step spin‐coating deposition using CsI and MAFA precursor solution (solution‐based, path 1) and powdered polycrystalline perovskites (mechanochemical, path 2). Reprinted with permission from Ref. [72]. Copyright, 2018 Elsevier.

Recently, a fast mechanochemical synthesis of Na_3_(VOPO_4_)_2_F, a nanocomposite cathode material has been reported, demonstrating high capacity and scalability up to 2 kg scale. This polyanionic cathode material shows an improved electrochemical performance for Na‐ion batteries. The nanocomposite exhibited a capacity of 142 mAh g^−1^ at 0.1 C, higher than its theoretical capacity of 130 mAh g^−1^ (see Figure 5a). This was attributed to the nano‐crystallization features and extra Na‐storage sites achieved during the solvent‐free mechanochemical synthesis process.^[66]^

In addition to these synthesis efforts, promising attempts have been made to use mechanochemistry for efficient and environmentally friendly battery recycling, offering easy operation, flexibility, and short processing times compared to traditional methods.^[73]^ Mechanochemical processes have been developed for extracting lithium from various cathode materials (LiCoO_2_, LiFePO_4_) in lithium‐ion batteries (LIBs), achieving recovery rates of up to 70 % without using corrosive leachates or applying high temperatures.^[74]^ The technique can be combined with hydrometallurgical processes and offers the potential for reagent regeneration.^[75]^ Furthermore, the selective recovery of critical elements (Li, Co, Ni, Mn) from end‐of‐life LIB cathode materials has been demonstrated using the deep eutectic solvent choline chloride‐formic acid at moderate temperatures.^[76]^ In this context, an environmentally friendly tandem approach for the selective recovery of lithium and simultaneous synthesis of the magnetic compound LiFe_5_O_8_ from spent LiFePO_4_ batteries has also been presented by mechanochemical processing (see Figure 5b).^[71]^

Mechanochemistry has also been applied to synthesise hybrid organic–inorganic metal halide perovskites (MHPs) for photovoltaic applications.[^77^, ^78^, ^79^] Mechanochemical processes proved to be effective especially synthesising multicomponent mixed cation and mixed halide perovskites. For example, the perovskite composition Cs_0.05_(MA_0.17_FA_0.83_)_0.9_Pb(I_0.83_Br_0.17_)_3_ (CsMAFA) has been extensively investigated for high‐efficiency perovskite solar cells.^[80]^ Typically, CSMAFA is synthesised using a one‐step spin‐coating process involving a solution of CsI in dimethylsulfoxide (DMSO) with a precursor solution of MA_x_FA_1‐x_Pb(I1‐xBr_x_)_3_. However, the use of other caesium halides such as CsBr or CsCl is limited due to their poor solubility in DMSO and dimethylformamide (DMF), hindering further advances in perovskite composition. To address this, researchers have developed a mechanochemical synthesis method by grinding the respective precursors in the correct ratio, which overcomes the solubility issues and allows for more versatile compositional engineering of perovskites (see Figure 5c).^[72]^ Furthermore, phase‐pure CsSnX_3_ (X=I, Br, Cl) perovskites have been prepared by ball milling respective metal salts.^[81]^ The mechanochemical synthesis of lead‐free perovskites can be upscaled to kilograms and used to prepare functional photodetector devices without any post‐synthesis treatment.

In the context of photovoltaic applications, also sulphides such as stannite (Cu_2_FeSnS_4_) and kesterite (Cu_2_ZnSnS_4_) have been synthesised by ball milling in an industrial setting.^[82]^ The authors report on the challenge of maintaining product purity which required the optimisation of grinding conditions such as grinding time and ball‐to‐powder ratio to ensure complete consumption of precursors.

The potential of mechanochemical synthesis for the preparation of hydrogen storage materials indicates significant advantages in terms of efficiency and cost‐effectiveness.^[83]^ Recently α‐AlH_3_ nanocomposites were produced via solid‐state mechanochemical reactions between LiH and AlCl_3_ in the presence of TiF_3_. The material achieved a maximum of hydrogen adsorption of 9.92 wt % at 160 °C.^[84]^ A quasi‐solid‐state template strategy to synthesise highly dispersed supported metal catalysts on nitrogen‐doped carbon, thereby improving the kinetics of MgH_2_‐based hydrogen storage materials has recently been provided.^[85]^ Moreover, a novel catalyst‐free method for synthesising lithium hydride (LiH) under mild conditions (room temperature, 30 bar H_2_ pressure), using organic solvents, has been developed. Their experimental results indicate that different organic solvents can effectively facilitate the hydrogenation of lithium metal. Acetone was found to produce lithium hydride of high purity (>98 %), which could significantly reduce production costs on an industrial scale.^[86]^ Furthermore, the mechanochemical synthesis of MOF‐5 exhibited enhanced hydrogen storage capabilities relative to solvothermal‐prepared counterparts. Researchers reported a 207 % increase in specific surface area and a 90.5 % improvement in maximum excess adsorption capacity at 77 K.^[87]^

## SDG 9: Industry, Innovation and Infrastructure

The objectives of SDG 9 have many synergies with the idea of a circular economy which also aims for more efficient production processes at reduced costs.^[88]^ To enable a circular economy, new business ideas are essential and are generated by the rise of innovative research through start‐ups and spin‐offs. Once these innovations are developed, their intellectual property needs to be protected to build a business case and attract investment. To stimulate the transfer of research to industry it is essential to implement effective policies and to make significant private and public investments.

The European Union is the spearhead of policymaking in creating a cost‐efficient and sustainable market and leading the way on the global stage with its European Green Deal and corporate sustainability strategies and measures. The objective of the Green Deal is to become the first carbon‐neutral continent by 2050,^[89]^ while corporate sustainability reporting (CSR) is a transparent tool for companies about their environmental, social, and governance impacts.^[90]^ The CSR and its content are strongly orientated on the SDGs and oblige companies to demonstrate to their customers, investors, regulators, and the public information regarding their carbon emissions and environmental footprint. For example, the use of natural resources, the production of waste, and their investment in clean production technologies. Despite the additional burden due to these reporting requirements, it also pushes chemical manufacturers to reflect their activities from a sustainable point‐of‐view and fine‐tune their business models. This includes the opportunity for companies on the market to utilise technology from the field of green chemistry such as mechanochemistry. Conversely, it also creates a marketplace for startups and spinoffs which make use of mechanochemical technologies. For them, the key to developing viable business models is to show improvements in the cost‐efficiency of those chemical processes that are traditionally labelled as being cost‐inefficient and that perform badly in terms of resource and chemical consumption.

**Figure 6:**
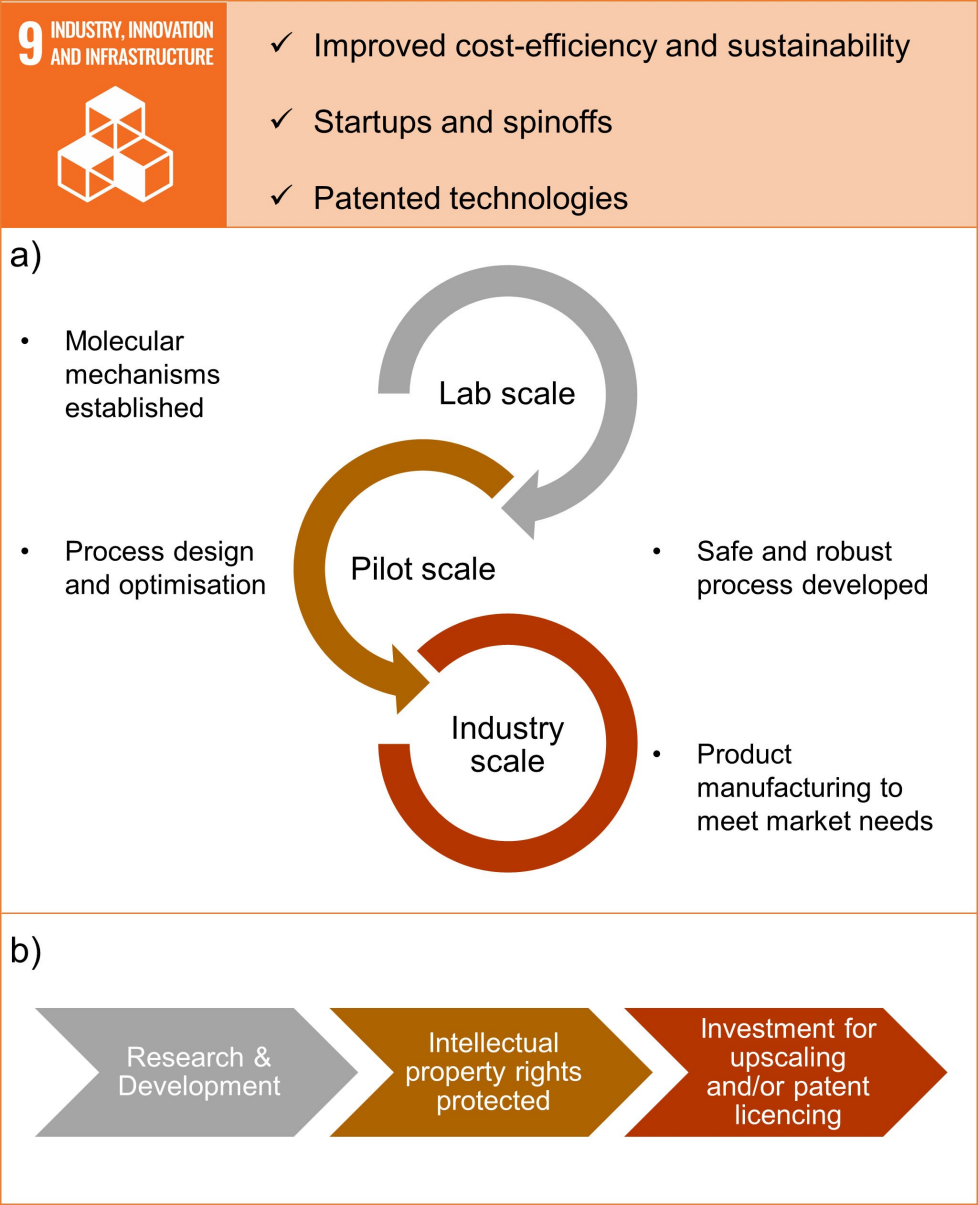
Examples of mechanochemistry‘s contributions to SDG 9: Industry, Innovation and Infrastructure. a) An idealised path from the laboratory to the industrial implementation. b) Simplified scheme of key steps in moving from the laboratory to the market.

Indeed, several such startups and small and medium‐sized enterprises (SMEs) based on mechanochemistry exist. For example, MOF Technologies, a company based in the UK, employs TSE for large‐scale preparation of MOF adsorbent materials. The company has recently received a Series A investment of over £10 million for its Nuada carbon capture technology.^[91]^ Matter from Germany offers chemical recycling of PET waste with their patented TSE revolPET® technology.^[92]^ In a continuous process, PET is mixed with NaOH and water to yield disodium terephthalate, which after workup results in terephthalic acid monomer that can be repolymerized into PET. Environmental Decontamination from New Zealand remedies environmental pollution, such as persistent organic pollutants or asbestos waste, with their patented continuous ball milling reactor.^[93]^ Several ongoing projects have shown their ability to degrade soil contaminants on a pilot scale. UK‐based Fluorok makes synthesis of value‐added fluorochemicals more sustainable.^[94]^ Their patented mechanochemical technology enables the direct activation of fluor‐containing minerals, such as fluorspar (CaF_2_), and their use as fluorinating agents. MechanoCross, a Japanese company, offers customised solutions for mechanochemical organic synthesis, encompassing laboratory‐scale and industrial‐scale applications.^[95]^ The innovation potential of mechanochemical technologies is also evident in the increasing number of patent applications directly based on mechanochemistry.^[11]^ However, the key issue for the business viability of mechanochemistry‐based startups and SMEs is upscaling and meeting the demands of the industrial sector (Figure 6).[^96^, ^97^] So far, no industrial‐scale plants are using innovative mechanochemical technologies, but this is likely to change in the coming years, driven at least by the international and European demand for more efficient and sustainable (intermediate) products.

## SDG 12: Responsible Consumption and Production

**Figure 7:**
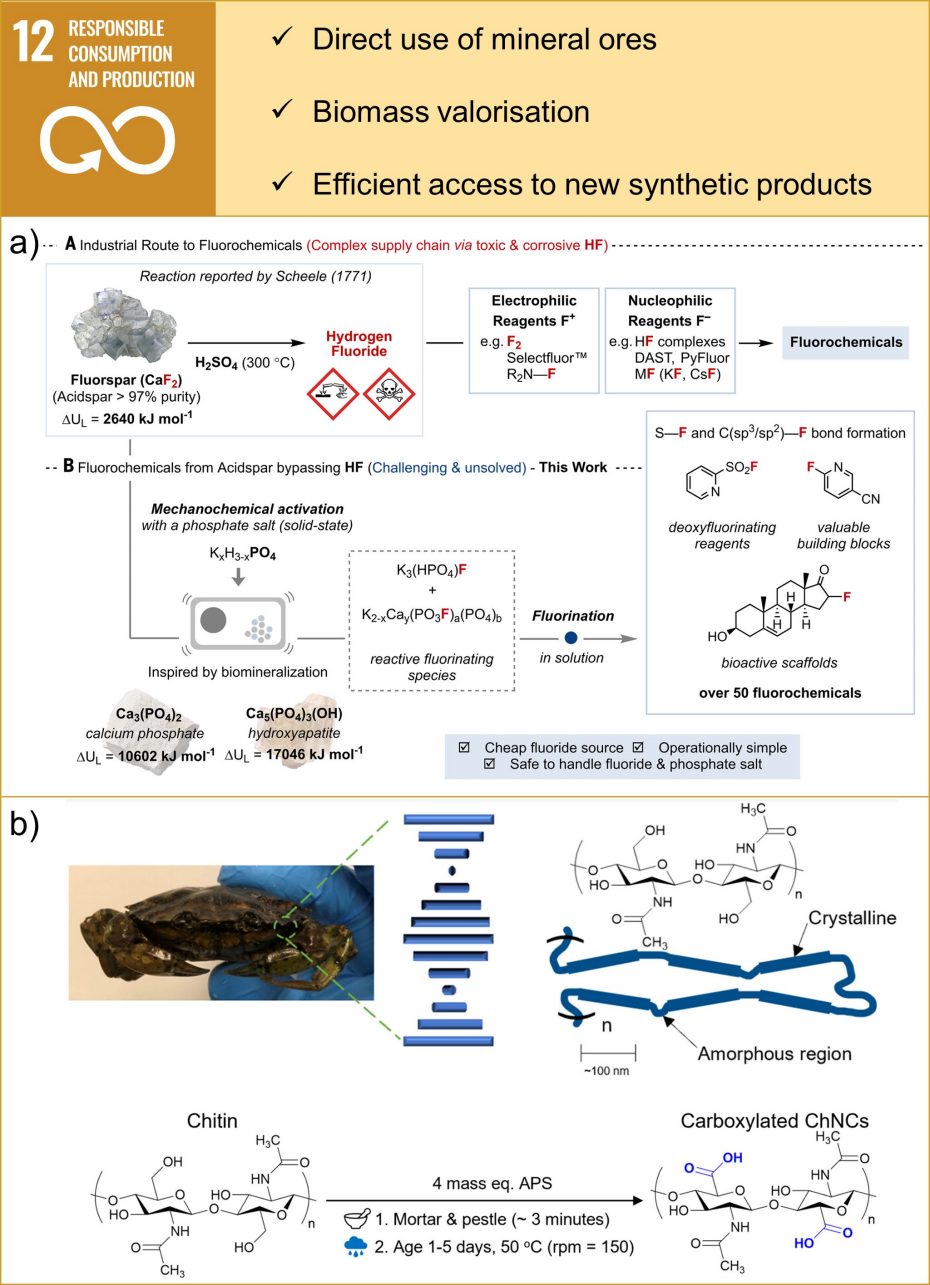
Examples of contributions of mechanochemistry towards SDG 12: Responsible Consumption and Production. a) The context of fluorochemicals production and mechanochemical activation of fluorspar. Adapted and reprinted from Ref. [100] with permission from AAAS. b) Supramolecular architecture of polysaccharides in green crab shells and reaction conditions leading to carboxylated chitin nanocrystals. From Ref. [120]. Copyright reproduced with permission from Wiley‐VCH Verlag GmbH & Co. KGaA, Weinheim.

Implementing sustainable consumption and production patterns is a task that is particularly dependent on chemistry and relies on the careful use of natural raw materials for the production of synthetic products. For example, metal catalysts are ubiquitous in chemistry and are typically extracted from mineral ores in chemical‐ and energy‐intensive production processes. However, direct catalysis by minerals is important and inspires scientists to mimic such processes.^[98]^ A recent study illustrates the potential of Earth‐abundant minerals to be directly used as catalysts in mechanochemical carbon‐carbon bond formation reactions.^[99]^ A broad substrate scope has been demonstrated for copper‐catalysed atom transfer radical cyclization reactions by using covellite (CuS) and oxidative coupling reactions by using vanadinite ([Pb_5_(VO_4_)_3_Cl]). Furthermore, a recent example demonstrates the use of fluorspar (CaF_2_) as a fluorinating agent to produce a wide range of fluorochemicals.^[100]^ Traditionally, insoluble fluorspar needed to be converted to toxic and corrosive HF using highly unsustainable processing (Figure 7a). However, researchers have developed a method that bypasses HF and relies on ball milling of fluorspar with dipotassium hydrogen phosphate salt. The reactions require a high input of mechanical energy and in 9 h yield a crystalline solid composed of K_3_(HPO_4_)F and K_2−x_Ca_y_(PO_3_F)_a_(PO_4_)_b_, called fluoromix, which can be used as a fluorinating agent.

Direct mechanocatalysis is another innovative approach that uses grinding equipment made of different materials that act as catalysts themselves.^[101]^ Such an approach drastically simplifies workup and product isolation. For example, milling jars and balls made of copper and palladium are highly efficient in Sonogashira[^102^, ^103^] and Suzuki coupling reactions.^[104]^ Other notable examples of cross‐coupling reactions include using insoluble aryl halides as reagents by high‐temperature ball milling.[^105^, ^106^] More generally, several classic organic reactions are currently experiencing a renaissance under mechanochemical conditions. For example, ball milling enables Grignard reactions in air^[107]^ and Birch reductions in minutes.[^108^, ^109^, ^110^] Furthermore, redox reactions using piezoelectric materials,[^111^, ^112^] synthesis of alkalides,^[113]^ and the production of reduced phosphorus compounds^[114]^ are among recent highlights in synthetic mechanochemistry.

Turning to natural resources, the valorisation of biomass waste has the potential to offer renewable feedstocks for many useful chemical processes. However, the extraction of valuable chemical building blocks from lignin, cellulose, hemicellulose, and chitin is hindered by their poor solubility and complex molecular structures. Mechanochemical processing not only overcomes these drawbacks^[115]^ but shows potential for scale‐up of biomass conversion with an increased energy efficiency.^[116]^ The acetylation behaviour of wood powder with acetic anhydride and pyridine using rod milling has recently been studied and shown to improve the thermal resistance of acetylated wood.^[117]^ Moreover, mechanochemical processing of technical lignin with sodium percarbonate and sodium hydroxide resulted in its partial depolymerisation and the increase of carbonyl functionality.^[118]^ As a result, processed lignin showed improved water adsorption properties. Additionally, continuous synthesis of nanoporous carbon materials by extrusion using lignin, urea, and potassium carbonate has been reported. The resulting products showed surface areas above 3000 m^2^ g^−1^ after pyrolysis and a potential to be used as supercapacitors.^[119]^

Until recently, selective access to nanocrystalline domains within the complex supramolecular structures of polysaccharides, having broad potential applications in the chemical industry, has eluded scientists who were primarily focused on extraction in bulk aqueous systems.^[120]^ A high‐humidity shaker ageing methodology, involving solvent‐free grinding of polysaccharides with ammonium persulfate followed by ageing under high humidity on a shaker plate, enabled efficient access to chitin nanocrystals (Figure 7b). High humidity allowed gradual uptake of water up to the deliquescence point and the formation of carboxylated chitin nanocrystals in 61 % yield starting from crab shells and soft wood pulp. A similar trend was reported in the case of mechanochemical hydrolysis of chitin over a carbon‐based catalyst with weak acid sites.^[121]^ Researchers demonstrated that planetary milling for a total of 12 h selectively hydrolyses chitin to chitin‐oligosaccharides in good yields. The reason for high selectivity in mechanochemical hydrolysis was demonstrated to be a preference for hydrolysis of large chitin molecules, which contrasts typical examples of oligomers to monomers hydrolysis in solution. That is important because the bioactive properties of oligosaccharides are associated with oligomers (starting from trimers) instead of monosaccharides. Another example of biomass valorisation is a simultaneous one where both polyvinyl chloride and eggshell are used as feedstocks to produce benign and soluble calcium chloride.^[122]^ Quantitative dechlorination was demonstrated on a laboratory scale with planetary milling and resulted in 55 % conversion upon upscaling to a 100 g scale using an eccentric vibratory ball mill.

Mechanochemistry is also valuable in the synthesis of polymers.^[123]^ For example, liquid‐assisted milling enabled the mechanochemical organocatalytic ring‐opening polymerisation of lactide to yield high molecular weight poly(lactic acid).^[124]^ Using longer milling times and larger milling balls together with higher milling frequencies was shown to increase the conversion rates, thus indicating a constructive transfer of mechanical energy to chain‐growth polymerisation of lactide, a renewable feedstock compound. Furthermore, anionic ring‐opening polymerisation of functional epoxide monomers was used to synthesise polyethers.^[125]^ Importantly, a higher melting point indicating stronger intermolecular interactions was shown to be a key parameter in predicting mechanochemical polymerisation reactivity. Furthermore, in stark contrast to solution reactivity, bulky functional monomers were shown to be more prone to polymerisation than linear ones.

## SDG 13: Climate Action

**Figure 8:**
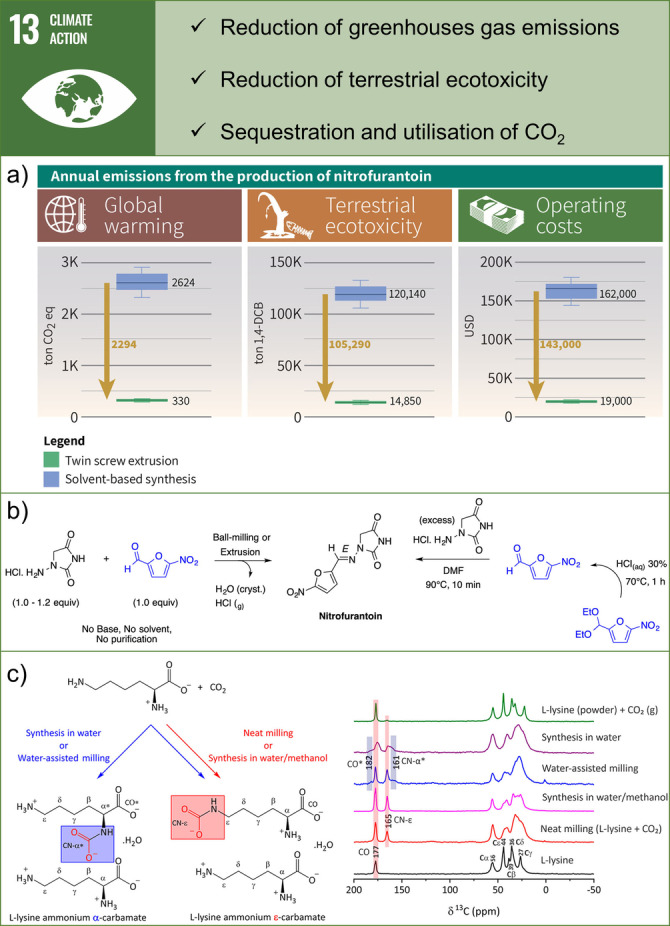
Examples of contributions of mechanochemistry towards SDG 13: Climate Action. a) Results of LCA for commercial‐scale nitrofurantoin API production using TSE and solvent‐based synthesis. Adapted with permission from Ref. [126]. Copyright 2022 American Chemical Society. b) Mechanochemical and solution‐based reaction conditions leading to nitrofurantoin. Adapted with permission from Ref. [127]. Copyright 2022 American Chemical Society. c) Products obtained by milling of L‐lysine with CO_2_ under different reaction conditions (left) and their corresponding ^1^H→^13^C cross‐polarization magic‐angle spinning NMR spectra. Adapted with permission from Ref. [130]. Copyright 2020 American Chemical Society.

Mechanochemistry meets SDG 13 by minimising solvent and reagents consumption, achieving energy savings through continuous processing, and significantly reducing greenhouse gas (GHG) emissions. Life cycle assessment (LCA) of API production by continuous TSE has been shown to considerably reduce CO_2_ emissions compared to solvent‐batch processing (Figure 8a).^[126]^ TSE production of nitrofurantoin, a mass‐produced API, requires significantly reduced input of chemicals, yields pure product, and eliminates the need for solvents (Figure 8b).

This saves more than 100,000 metric tons of toxic emissions (measured in 1,4‐dichlorobenzene equiv.) entering terrestrial organisms and plants, enabling substantial cost reduction. Additionally, TSE production of nitrofurantoin decreases GHG emissions by approximately 2,300 metric tons of CO_2_ equivalents annually, demonstrating the enormous environmental benefits of using mechanochemistry for manufacturing APIs.

Furthermore, the greenness of mechanochemical nitrofurantoin production has been quantified using the DOZN 2.0 tool, a universal green chemistry evaluator based on the 12 principles of green chemistry.^[127]^ DOZN 2.0 calculates scores of processes on a scale of 0 to 100, where 0 indicates the greenest process. The study provides the quantitative greenness assessment for batch and continuous mechanochemical processes of nitrofurantoin production carried out with different mechanochemical techniques. The scores show compliance of all mechanochemical processes with the 12 principles of green chemistry and emphasise the continuous TSE process as the greenest.

Importantly, mechanochemical production processes often require product isolation and purification using solvents. To determine the environmental impact of pigment black 31 (P.B. 31) production using TSE, a LCA has been carried out and compared to the solvent‐batch process.^[128]^ The TSE process achieves higher yields and only requires methanol washing, while the solvent‐batch process requires dimethylformamide and methanol to remove the unreacted reagents. As a result, the estimated scale‐up production of P.B. 31 using the TSE process has approximately an order of magnitude lower environmental impact compared to the solvent batch process.

In addition, mechanochemistry has been shown to reduce huge CO_2_ emissions from cement production by substituting high CO_2_‐emitting cement clinker with an alternative alkali‐activated binder.^[129]^ This is achieved by ball milling of CaCO_3_ with Na_2_SiO_3_ at room temperature, which leads to the formation of reactive amorphous aNaSiCC intermediate in a salt metathesis reaction.

Additionally, mechanochemistry can also be used to sequester CO_2_ by using it as an abundant and inexpensive source of carbon to produce a range of valuable chemicals. One example involves the conversion of CO_2_ into L‐lysine ammonium ϵ‐carbamate by solvent‐free ball milling with L‐lysine (Figure 8c).^[130]^ Compared to traditional solution synthesis in water, which yields a mixture of carbamates (at the α‐NH_2_ and ϵ‐NH_2_ positions, Figure 8c right, purple spectrum), neat milling yields only ϵ‐carbamate (Figure 8c right, red spectrum).

Another example of CO_2_ use involves ball milling CaC_2_ with CO_2_ to produce oxygenated alkynyl carbon material with supercapacitive performance.^[131]^ The key reaction step is the mechanochemical activation of CaC_2_ whose reactivity is typically limited by highly stable crystalline structure. Upon mechanical treatment of CaC_2_, the activation energy is significantly reduced, and results in almost quantitative product yield and complete CO_2_ consumption. Furthermore, biguanidine is an active species that can be used for CO_2_ capture and conversion to biguanidinium carbonate and bicarbonate hydrogen‐bonded networks by mechanochemistry.^[132]^ In particular, liquid‐assisted grinding (LAG) reactions show inverse selectivity for different carbonate and bicarbonate networks when using the same solvents as in solution synthesis. Interestingly, CO_2_ can also be used as a C1 synthon in mechanochemical liquid‐assisted Grignard reactions with LiOH.^[133]^

Mechanochemistry also serves as a green tool in designing new catalysts for carbon capture and utilization. For example, mechanosynthesized crystalline and amorphous ZnCu‐MOF‐74 have been tested as heterogeneous catalysts for the hydrogenation of CO_2_ to methanol.^[134]^ Interestingly, despite its collapsed structure and very low porosity, amorphous ZnCu‐MOF‐74 shows better catalytic activity and selectivity towards methanol production when compared to crystalline ZnCu‐MOF‐74. The defects introduced by ball milling change the morphology of the catalyst and make the Zn and Cu active sites in amorphous ZnCu‐MOF‐74 more accessible to CO_2_. Moreover, the amorphous ZnCu‐MOF‐74 catalyst even shows similar selectivity towards methanol compared to commercial Cu/ZnO/Al_2_O_3_ catalyst.

## SDG 14: Life Below Water & SDG 15: Life on Land

Polymers have many interesting properties that have made them indispensable in our daily lives and are cheap to produce using fossil fuels. However, due to increased production and single‐use plastics, we are now facing plastic pollution. The main problem is that traditional recycling technologies cannot process most waste streams, so plastic waste is either incinerated or landfilled in an unsustainable way.^[135]^ Chemical recycling is a promising technology that has the potential for closed‐loop processing of most plastic waste streams but is limited by high costs.^[136]^ Driven primarily by the reduction of energy consumption with ambient reaction conditions and the reduction of waste by eliminating solvents in a synthesis step, mechanochemistry has the potential to significantly reduce the processing costs typically associated with chemical recycling.[^137^, ^138^]

**Figure 9:**
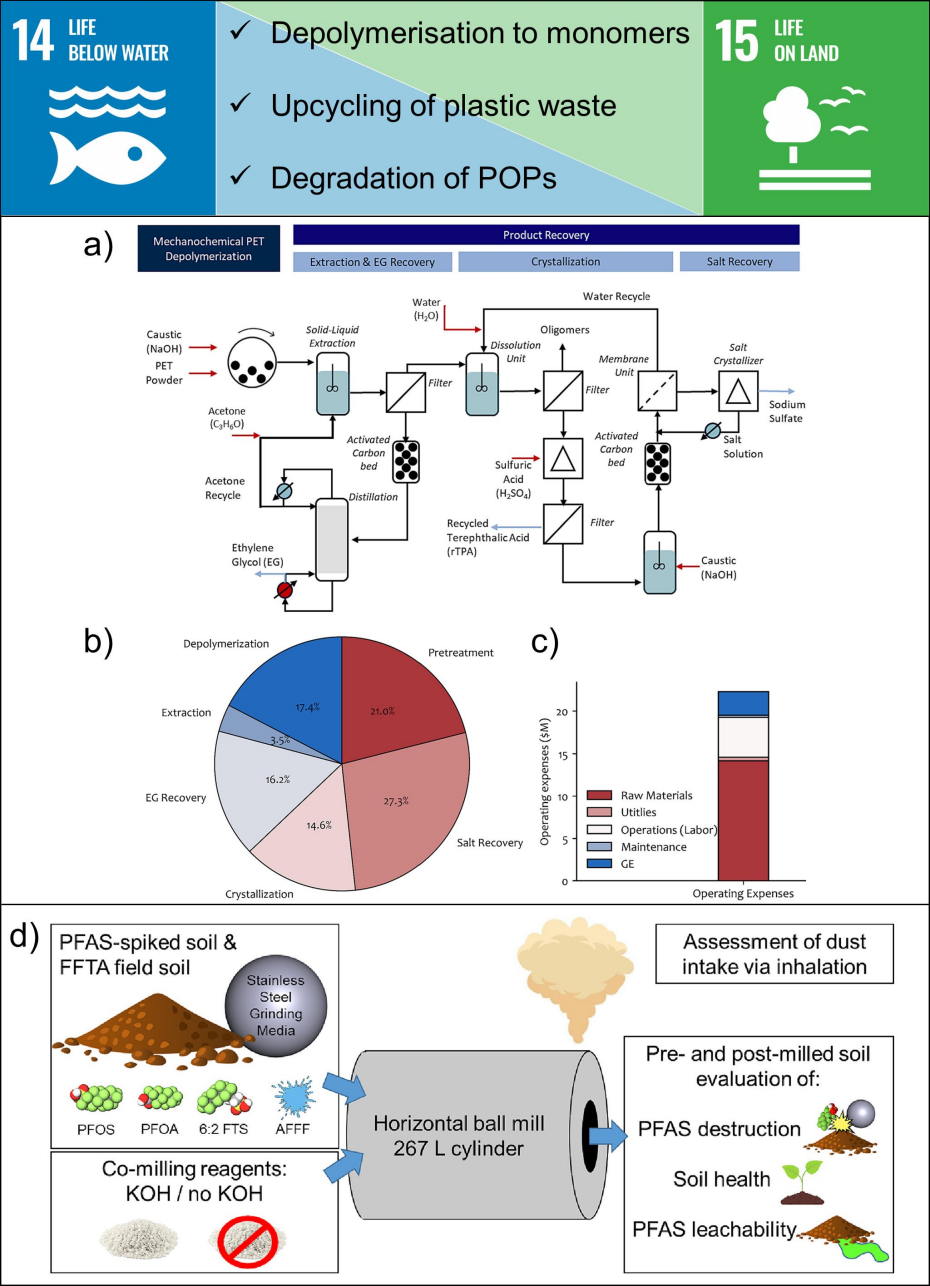
Examples of contributions of mechanochemistry towards SDG 14: Life Below Water and SDG 15: Life on Land. a) Process for the chemical recycling of PET based on mechanochemical depolymerisation with sodium hydroxide. b) Capital costs broken down into different process parts. c) Breakdown of operating costs. Adapted with permission from Ref. [141]. Copyright (2024) Elsevier. d) Mechanochemical degradation of per‐ and polyfluoroalkyl substances in soil using an industrial‐scale horizontal ball mill. Reprinted with permission from Ref. [152]. Copyright (2024) Elsevier.

For example, alkaline hydrolysis of polyethylene terephthalate (PET) by ball milling results in a quantitative depolymerisation to disodium terephthalate, which upon acid extraction yields recycled terephthalic acid (rTPA) monomers.[^139^, ^140^] The use of a larger grinding ball and increased mechanical energy transfer through impact results in faster depolymerisation, which can be completed within 20 min of reaction time. Importantly, this approach allows the use of PET textile waste and coloured PET waste as feedstock without compromising the conversion rate of the depolymerisation reaction and the yield of high‐purity rTPA.^[139]^ In addition, process development and techno‐economic analysis for mechanochemical recycling of poly(ethylene terephthalate) based on depolymerisation with sodium hydroxide clearly demonstrate the economic viability of this technology (Figure 9a–c).^[141]^ Importantly, the minimum selling price (MSP) for the mechanochemical depolymerisation technology is highly competitive and is projected at $0.99/kg rTPA, which is almost the same as the recycled dimethyl terephthalate (rDMT) from methanolysis ($0.96/kg), but lower than virgin TPA from fossil fuels ($1.14/kg) and rTPA from enzymatic depolymerisation ($1.93/kg).

Another mechanochemical approach to PET depolymerisation has recently been reported using direct methanolysis in planetary mills to produce rDMT. Interestingly, this approach is also highly efficient in the depolymerisation of polycarbonate and poly(lactic acid).^[142]^ Mechanochemical enzymatic depolymerization of PET has also been reported.^[143]^ Furthermore, the hydrogenation of different carbon substrates into small molecule hydrocarbons has been achieved by ball milling.^[144]^ For example, planetary milling of plastics, biomass, coal, and even diamond under 170 bar of H_2_ results in mixtures of C_1_−C_4_ products. The hydrogenation efficiency improves with increasing input of mechanical energy and follows a radical‐based mechanism dependent on metal catalysts. Similarly, the mechanical energy provided by ball milling generates homolytic cleavage of the commodity polymer chains, initiating radical chain dehalogenation or fluorination reactions.^[145]^

Per‐ and poly‐fluoroalkyl substances (PFAS) are man‐made organic compounds with fully or partially fluorinated chemical structures. Due to their water‐, oil‐ and dirt‐resistant properties, they are used in various consumer and industrial products.^[146]^ However, their persistence and health effects associated with exposure to these chemicals are reasons to be classified as persistent organic pollutants (POPs) and make their environmental remediation a top priority. Mechanochemistry contributes to SDG 14 and 15 by helping in PFAS remediation in aquatic and solid environments.^[147]^ Regarding aquatic environments, mechanochemistry allows the green synthesis of various adsorbents for PFAS removal, while in solid environments, mechanical energy directly leads to PFAS degradation.

Remediation of solid PFAS by mechanochemistry is among the most promising and sustainable approaches that typically involves high‐energy ball milling of PFAS with co‐milling reagents. The most used include metal oxides^[148]^ and hydroxides,[^149^, ^150^, ^151^, ^152^] oxidants,^[153]^ zero valent metals,^[154]^ and neutral compounds such as SiO_2_.^[155]^ Regarding the efficiency of PFAS degradation, the emphasis is on achieving complete degradation (mineralisation to inorganic carbon and fluoride) and minimising waste generation.

For instance, mechanochemical destruction of PFAS involves grinding perfluorooctanoic acid (PFOA) and perfluorooctane sulphonic acid (PFOS), two common PFAS found in the environment, with KOH in a planetary ball mill.^[149]^ The findings show that mechanical force causes C−F bond cleavage while co‐milling with KOH significantly enhances the degree of defluorination, which reaches up to 96.7 % after 4 hours of milling. Also, ball milling with KOH enables PFAS degradation in water‐saturated sands and PFAS‐contaminated soils from firefighting training areas.^[151]^ However, the mass ratio between KOH and PFAS affects the defluorination efficiency which decreases when using lower mass ratios. In addition, a reaction with KOH produces toxic KF. Consequently, the excess of KOH used to achieve complete defluorination and resulting KF require further treatment before being disposed of in the environment thus reducing the cost‐efficiency of the process.

Furthermore, researchers demonstrated that pure mechanical energy can break down PFAS in impacted soils, while destruction of PFAS present in liquid samples such as commercial firefighting foams can be achieved by the addition of quartz sand.^[155]^ In both cases, mechanical activation of quartz sand, which is also present in contaminated soil, generates silyl and siloxy mechanoradicals that act as initiators and propagators of C−F and C−C bond cleavage.^[156]^

Interestingly, mechanochemistry can also be used for PFAS valorisation by converting them into economically important products such as organofluorine blocks^[157]^ or luminescent materials.^[144]^

In this regard, a ball milling of PFOA with Al_2_O_3_ at room temperature and pressure results in partial defluorination of PFOA and formation of 1H‐perfluorohept‐ene.^[157]^ The major advantage of mechanochemistry is enabling simple, fast, and selective defluorination of perfluoroalkane into perfluoroalkenes, which is otherwise a very challenging process and often results in the formation of aromatic compounds.

## Outlook

In recent years, there has been a surge in research into mechanochemistry and its alignment with the UN SDGs, particularly in areas such as environmental remediation, energy materials, and sustainable consumption and production. This growth is being driven by the effectiveness of mechanochemistry compared to other methods. By using mechanical forces to drive chemical reactions, mechanochemistry reduces the need for hazardous solvents, minimises waste, and improves energy efficiency. Green metrics such as atom economy, E‐factor, process mass intensity, and LCA help assess the environmental impact of these processes, ensuring they are both scientifically robust and environmentally friendly. As the field further evolves, mechanochemistry‘s disruptive potential to support a sustainable and circular economy is becoming increasingly clear.^[158]^ Combined with its cost‐effectiveness and investments in clean technologies, mechanochemical advances are poised to address major societal challenges.

## Conflict of Interests

The authors declare no conflict of interest.

1

## Biographical Information

*Tomislav Stolar is an Adolf Martens Postdoctoral Fellow at the Federal Institute for Materials Research and Testing (BAM) in Berlin. He obtained his PhD from the University of Zagreb, Croatia. His research broadly covers the field of mechanochemistry with a focus on mechanochemical depolymerisation of plastic waste and research transfer to the industry. He won highly competitive Falling Walls Berlin‐Adlershof pitching competition and presented about mechanochemistry at Falling Walls Science Summit 2023*.

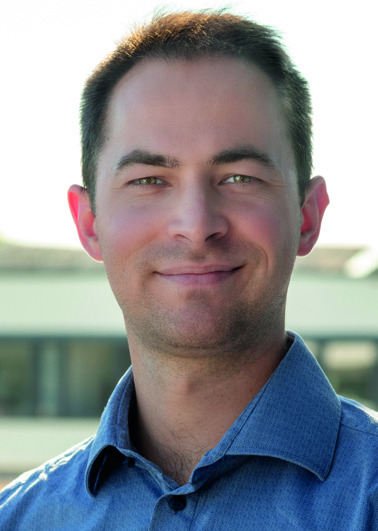

## Biographical Information

*Moritz‐Caspar Schlegel is a researcher at the Federal Institute for Materials Research and Testing (BAM) in Berlin. He holds a PhD from the Humboldt University of Berlin. His research focuses on eco‐design, energy efficiency, and circular economy initiatives. He coordinates circular economy projects at BAM and contributes to the development of sustainable product regulations in Europe, in particular the Ecodesign for Sustainable Products Regulation (ESPR)*.

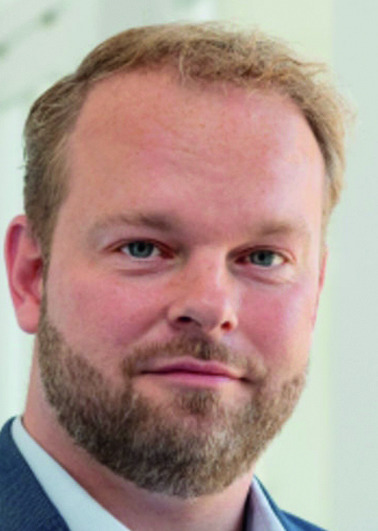

## Biographical Information

*Jasna Alić is an Adolf Martens Postdoctoral Fellow at the Federal Institute for Materials Research and Testing (BAM) in Berlin. She holds a PhD from the University of Zagreb, Croatia, and is known for her innovative work in the development of environmentally friendly chemical processes. Her research focuses on mechanochemistry, including sustainable synthesis of materials and degradation of environmental pollutants*.

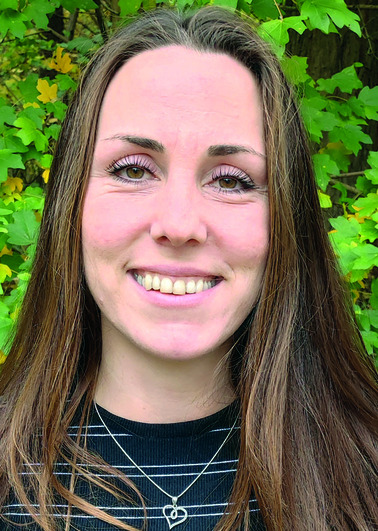

## Biographical Information

*Franziska Emmerling is Head of the Materials Chemistry Department at the Federal Institute for Materials Research and Testing (BAM) in Berlin. Her research covers a broad range of materials chemistry, with a particular focus on mechanochemistry and in situ investigations of crystallisation processes. Her recent work involves the development of innovative methods for mechanochemical synthesis, and the application of time‐resolved experiments to advance sustainable chemical processes and improve the efficiency of materials synthesis*.

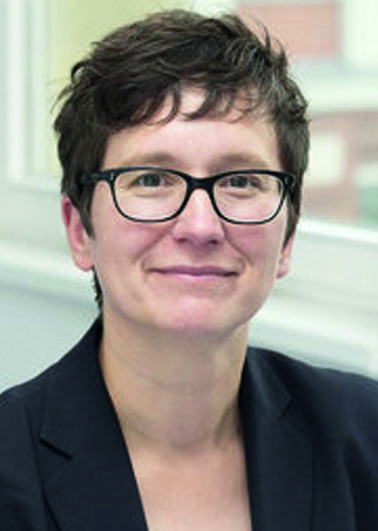

## Data Availability

The data that support the findings of this study are available from the corresponding author upon reasonable request.
